# Syntheses of Four Enantiomers of 2,3-*Diendo*- and 3-*Endo*-aminobicyclo[2.2.2]oct-5-ene-2-*exo*-carboxylic Acid and Their Saturated Analogues

**DOI:** 10.3390/molecules181215080

**Published:** 2013-12-06

**Authors:** Márta Palkó, Mikko M. Hänninen, Reijo Sillanpää, Ferenc Fülöp

**Affiliations:** 1 Institute of Pharmaceutical Chemistry, University of Szeged, Szeged H-6720, Eötvös utca 6, Hungary; E-Mail: palko@pharm.u-szeged.hu; 2 Department of Chemistry, University of Jyväskylä, Turku FIN-40014, Finland; E-Mails: mikko.m.hanninen@jyu.fi (M.M.H.); resillan@jyu.fi (R.S.); 3 Stereochemistry Research Group of the Hungarian Academy of Sciences, University of Szeged, Szeged H-6720, Eötvös utca 6, Hungary

**Keywords:** bicyclic β-amino acid derivatives, constrained chiral β-amino acids, resolution, continuous flow hydrogenations

## Abstract

Ethyl 2,3-*diendo*-3-aminobicyclo[2.2.2]oct-5-ene-2-carboxylate ((±)-**1**) was resolved with *O*,*O*'-dibenzoyltartaric acid via diastereomeric salt formation. The efficient synthesis of the enantiomers of 2,3-*diendo*-3-aminobicyclo[2.2.2]oct-5-ene-2-carboxylic acid ((+)-**7** and (–)-**7**), 3-*endo*-aminobicyclo[2.2.2]oct-5-ene-2-*exo*-carboxylic acid ((+)-**5** and (–)-**5**), *cis*- and *trans*-3-aminobicyclo[2.2.2]octane-2-carboxylic acid ((+)-**6**, (–)-**6**, (+)-**8** and (–)-**8**) was achieved via isomerization, hydrogenation and hydrolysis of the corresponding esters (–)-**1** and (+)-**1**. The stereochemistry and relative configurations of the synthesized compounds were determined by NMR spectroscopy (based on the ^3^*J*(H,H) coupling constants) and X-ray crystallography.

## 1. Introduction

In the past decade, the number of investigations on β-amino acids, in both racemic and optically active forms, has risen exponentially as a consequence of their increasing chemical and biological importance. β-Amino acids and their derivatives possess noteworthy pharmacological effects. For example, the first natural alicyclic β-amino acid, (1*R*,2*S*)-2-aminocyclopentanecarboxylic acid (cispentacin) exhibits antifungal activity [1,2,3,4]. Alicyclic β-amino acids can also be used as building blocks of modified analogues of pharmacologically active peptides [5,6,7,8,9,10].

While a range of stereoselective syntheses of enantiomerically enriched small (three- or four-membered: [11,12,13] and medium (five- to eight-membered) cyclic [14,15,16,17] and sterically hindered bicyclic norbornane and norbornene β-amino acids [18,19] or their derivatives have been reported, relatively few examples cocerning the synthesis and applications of enantiomerically enriched bicyclo[2.2.2]octane β-amino acid derivatives have been described [20,21,22,23,24].

Many attractive techniques are available for the production of enantiomerically pure amino acids. Resolution via diastereomeric salt formation, for instance, is still useful for the production of enantiomerically pure compounds on a laboratory scale. Resolution with an equivalent of the resolving agent (Pasteur’s method) is the most convenient in work with small amounts of the racemate (DL). In resolution with one equivalent of the resolving agent (R), diastereomers are separated (DL+2R → DR+LR), while when half an equivalent of resolving agent and half an equivalent of achiral acid or base (I) are used (Pope-Peacheay’s method [25,26,27,28], a diastereomeric salt is separated from a salt with an achiral agent (DL+R+I → DR+LI). The role of the achiral acid or base is to form a highly soluble salt with the enantiomer remaining in the solution. If there is a large difference in stability of the resulting diastereomeric salt and the enantiomer remaining in solution, the resolutions may be performed with half an equivalent of resolving agent and the achiral auxiliary material is omitted (DL+R → DR+L). A synthesis of racemic 2,3-*diendo*-3-aminobicyclo[2.2.2]oct-5-ene-2-carboxylic acid has been published [29], but its C-2 epimer and their enantiomers have not yet been examined. Our present aim was to synthetize of the title alicyclic β-amino acid enantiomers. This report focuses on the resolution of racemic 2,3-*diendo*-3-aminobicyclo[2.2.2]oct-5-ene-2-carboxylic acid derivatives by diastereomeric salt formation.

## 2. Results and Discussion

To find the most suitable resolving agent for (±)-**1**, three kinds of commercially available acidic resolving agents were examined. The solvent in each resolution experiment was EtOH. We first used half an equivalent of *L*-tartaric acid, and *S*-mandelic acid as the resolving agent for (±)-**1**, but surprisingly poor results were obtained. We next focused our attention on *O*,*O*'-dibenzoyltartaric acid (DBTA) as a resolving agent which is often used in gram-scale productions. In the first experiments of the Pope-Peacheay resolutions, 0.7 equivalent of aqueous HCl (achiral acid), 0.5 equivalent of DBTA as resolving agent and 1 equivalent of (±)-**1** were mixed with the solvent, and the mixture was heated at 70 °C. The solution was subsequently stirred and further cooled to room temperature. After stirring for 2 h, the solids were collected. Finally, this protocol was repeated without HCl. As the ^1^H-NMR spectra of the salt-pairs were identical, the diastereomeric purity of the salt obtained in each resolution experiment was determined as the diastereomeric excess (*de*%), based on the enantiomeric excess (*ee*%) of **1** in the salt. The experimental results are listed in Table 1.

**Table 1:** Resolutions of (±)-**1** with chiral acids.

Entry	Resolving agent	Yield (%)	*de* ^b^ (%)	S factor ^c^
1	*L*-tartaric acid	23	48	0.22
2	*S*-mandelic acid	30	45	0.27
3	L-DBTA ^a^	47	70	0.66
4	L-DBTA	45	84	0.76

^a^ In the presence of 0.7 equivalent of aq. HCl; ^b^ The *de* of the salt was determined via the *ee* of ester **1** liberated from the salt; ^c^ The efficiency factor S is used to indicate how effective a resolution is: S-factor = 2 × yield × *de* [30].

**Scheme 1:**
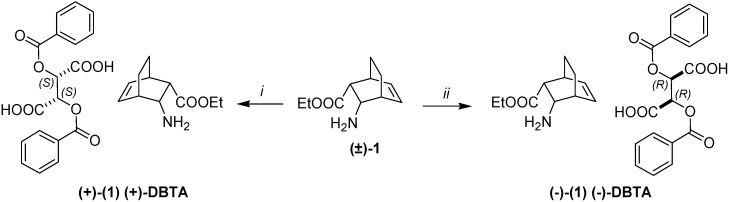
Resolution of ester **1** with DBTA.

Recrystallization of the L-DBTA salt containing (+)-**1** with 84% *de* in EtOH furnished enantiomer (+)-**1** with 96% *de*. Similarly, enantiomer (–)-**1** with D-DBTA was obtained from the mother liquor in 98% *de* (Scheme 1).

*Reagents and Conditions*: (*i*) (+)-DBTA, EtOH, 70 °C; (*ii*) (−)-DBTA, EtOH, 70 °C.

The C-2 isomerization of (–)-**1** with NaOEt in EtOH at 70 °C resulted in amino ester (+)-**2**. Compounds (–)-**1** and (+)-**2** were also transformed into amino ester enantiomers (–)-**3** and (–)-**4** with H_2_ in the presence of Pd/C. Continuous flow hydrogenations were carried out in a ThalesNano H-cube™ system. For each run, 150 mg of catalyst was added to in a tubular catalyst cartridge with an inner diameter of 4 mm and a length of 30 mm. The catalyst was rinsed for 0.5 h with a flow of EtOH 1 mL min^−1^, followed by pretreatment with H_2_ for 0.5 h in the same solvent. The reactant was dissolved in EtOH and this solution was delivered to the hydrogenation system via a conventional HPLC pump, through the mixer of the apparatus, where H_2_ was mixed into the liquid flow under a pressure of 1 MPa. The catalyst cartridge holder was equipped with an externally controlled cooling jacket. The mixture was pumped through the catalyst bed so as to obtain an ascending flow of the reaction components. After one reaction cycle, samples of 1 mL were taken from the product flow and analysed by GC analysis: complete conversion was observed.

**Scheme 2:**
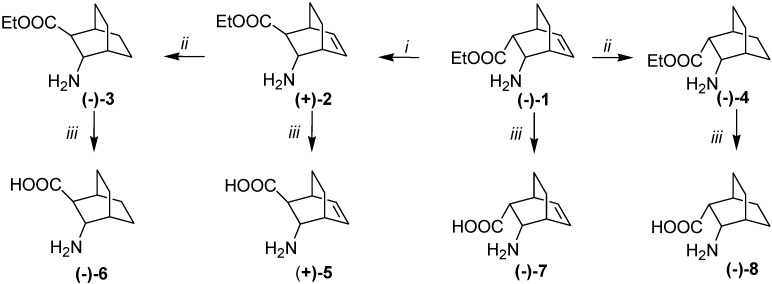
Synthesis of amino acid enantiomers (+)-**5**, (–)-**6**, (–)-**7** and (–)-**8**.

When subjected to microwave irradiation in H_2_O at 150 °C for 1 h, ester (–)-**1**, (+)-**2**, (–)-**3**, and (–)-**4** gave amino acids (+)-**5**, (–)-**6**, (–)-**7** and (–)-**8** (Scheme 2). All the reactions were first optimized for the racemic compounds. The whole syntheses were repeated, starting from racemic amino ester (±)-**1** and enantiomer (+)-**1**.

*Reagents and Conditions*: (*i*) NaOEt, EtOH, 70 °C; (ii) Pd/C, H_2_; (iii) MW, H_2_O, 100 °C.

**Scheme 3:**
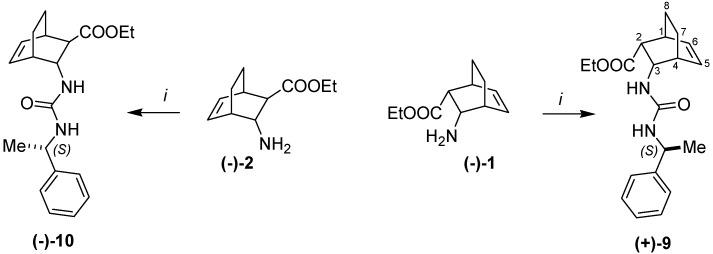
Synthesis of ureas (+)-**9** and (–)-**10**.

**Figure 1:**
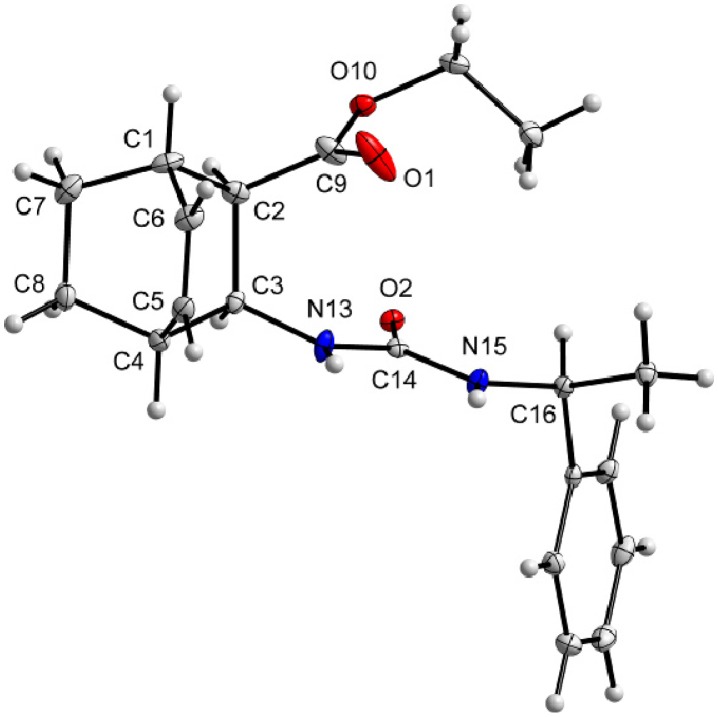
Molecular structure of carbamate (+)-**9**. Thermal ellipsoids have been drawn at the 20% probability level.

**Figure 2:**
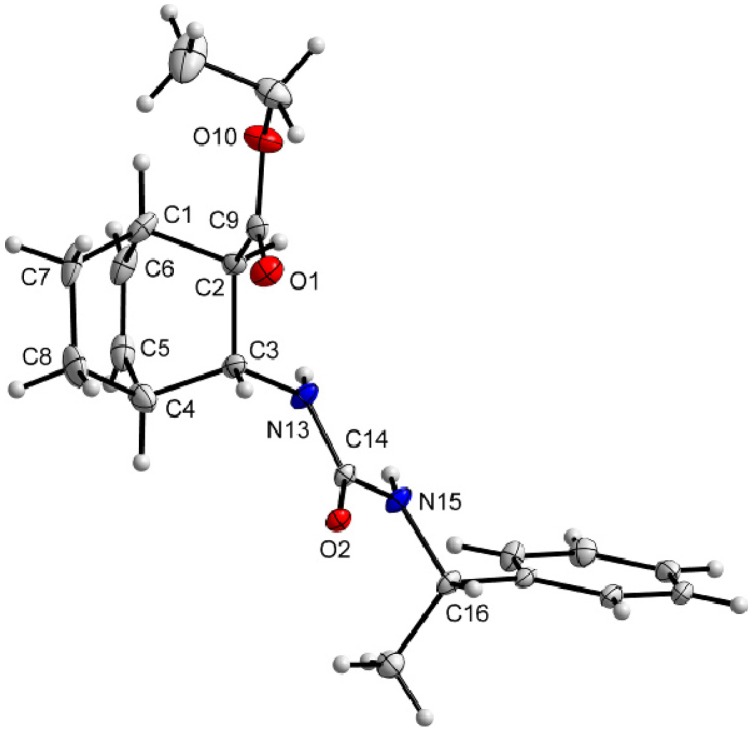
Molecular structure of carbamate (–)-**10**. Thermal ellipsoids have been drawn at the 20% probability level.

X-ray investigations revealed the absolute configurations of (–)-**1** and (–)-**2**. Amino esters (–)-**1** and (–)-**2** were transformed into ureas (+)-**9** and (–)-**10** upon reaction with (*S*)-(–)-α-methylbenzyl isocyanate (Scheme 3). The X-ray structures clearly showed the 1*S*,2*R*,3*S*,4*R* configuration of (–)-**1** (Figure 1) and the 1*R*,2*R*,3*R*,4*S* configuration of (–)-**2** (Figure 2).

*Reagents and Conditions*: (*i*) (*S*)-(‒)-α-methylbenzyl isocyanate, Et_2_O, rt.

## 3. Experimental

### 3.1. General

The chemicals were purchased from Aldrich (Budapest, Hungary) or Fluka (Budapest, Hungary) The ^1^H-NMR spectra were recorded at 400 MHz and the ^13^C-NMR spectra at 100 MHz on a Bruker AM 400 spectrometer at ambient temperature (27 °C) in DMSO-*d*_6_. Chemical shifts are given in δ (ppm) relative to TMS as internal standard. Elemental analyses were performed with a Perkin-Elmer CHNS-2400 Ser II Elemental Analyzer. Melting points were measured with a Hinotek X-4 melting point apparatus and are uncorrected. Optical rotations were measured with a Perkin-Elmer 341 polarimeter. Microwave reactions were performed in a CEM Discover LabMate MW reactor. Continuous hydrogenations were carried out in an H-Cube high-pressure continuous-flow system purchased from Thales Nanotechnology Inc. Racemic ethyl 2,3-*diendo*-3-aminobicyclo[2.2.2]oct-5-ene-2-carboxylate (±)-**1** was prepared by a literature method [29]. The *ee* values for the esters (–)-**1**, (+)-**1**, (‒)-**4** and (+)-**4** were determined after acylation with Ac_2_O in the presence of base by using Perkin-Elmer GC instrumentation equipped with CP-Chirasil L-Val columns [31]. All analyses for derivatized amino esters were performed with N_2_ as carrier gas; the column temperature and inlet pressure were adjusted to an optimized program. The injector and detector temperature were set at 250 °C and 270 °C, respectively. The *ee* values of **2** and **3** were determined by HPLC, using a Daicel Chiralcel ODH column. Samples were prepared for *ee* determination by HPLC analysis as follows: (+)-**2**·HCl, (–)-**2**·HCl, (+)-**3**·HCl or (–)-**3**·HCl (10 mg) was placed in a vial, to which 1 M aqueous NaOH (1 mL), toluene (1 mL) and benzoyl chloride (15 μL) were added. The mixture was allowed to stand for 15 min at room temperature, during which *N*-benzoylation proceeded to yield *N*-benzoyl-**2** or *N*-benzoyl-**3**. After separation, drying and evaporation of the toluene layer, the residue was dissolved in 2 mL a mixture of *n*-hexane and IPA (8:2) and the insolubles were removed by filtration. The filtrate (2 μL) was injected into the HPLC apparatus. The analytical conditions were as follows: (a) eluent: a mixture of *n*-hexane and IPA (8:2), flow rate: 0.25 mL min^−1^, detection at 233 nm, retention times: (–)-**2**: 26.1 min, (+)-**2**: 34.3 min; (b) a mixture of *n*-hexane and IPA (95:5), flow rate: 0,25 mL min^−1^, detection at 233 nm, retention times: (–)-**3**: 33.48 min, (+)-**3**: 35.2 min.

### 3.2. Determination of de Values of O,O'-dibenzoyltartaric Acid (DBTA) Salts of **1**

The *de* values of the salts were determined from the *ee* values of esters **1**. Samples were prepared for *ee* determination by GC analysis as follows: diastereomeric salt (5 mg) was dissolved in CH_2_Cl_2_ (50 μL). The sample was acylated with Ac_2_O (15 μL) and a mixture of 4-dimethylaminopyridine (DMAP) and pyridine (1:9) (15 μL) was added. After shaking for 2–3 s, the derivatized samples were analysed on GC CP-Chirasil L-Val columns (30 m) with the temperature held at 140 °C for 5 min and then raised to 160 °C at 10 °C min^−1^, flow rate: 2 mL min^−1^, retention times: (–)-1: 6.96 min, (+)-1: 7.11 min.

### 3.3. A Typical Resolution Procedure

A mixture of racemic ester base **1** (10.98 g, 5 mmol), (–)-DBTA (0.94 g, 2.5 mmol) and EtOH (9 mL) in a 50 mL flask was heated under stirring at 70 °C to obtain a clear solution. The solution was gradually cooled to 20 °C during about 30 min, and the precipitated diastereomeric salt was filtered off and washed with Et_2_O (10 mL) to yield white crystals, which were dried at room temperature to afford the (1*S*,2*R*,3*S*,4*R*)-(–)-1.(–)-DBTA salt (0.92 g, *de* 84%). The product was recrystallized from EtOH (yield 0.7 g, *de* 96%). The mother liquor was evaporated down and, 2 N NaOH solution (10 mL) was added to the residue. The mixture was extracted with CH_2_Cl_2 _(3 × 20 mL). The combined organic phase was dried (Na_2_SO_4_) and the solvent was evaporated off. (+)-DBTA (0.48 g, 1.3 mmol) and EtOH (6 mL) were added to the ester residue (+)-**1** (0.5 g, 1.6 mmol, *ee* 57%) followed by heating under stirring at 70 °C to give a clear solution. The solution was gradually cooled to 20 °C during about 30 min, and the precipitated diastereomeric salt was filtered off and washed with Et_2_O (10 mL) to yield white crystals, which were dried at room temperature to afford the (1*R*,2*S*,3*R*,4*S*)-(+)-**1**.(+)-DBTA salt (0.7 g, *de* 92.5%). The crystals were recrystallized from EtOH (yield 0.5 g, *de* 98%)

*Ethyl (1S*,*2R*,*3S*,*4R)-3-aminobicyclo[2.2.2]oct-5-ene-2-carboxylate* (–)-**1**. (–)-**1**·(–)-DBTA salt, mp 186–188 °C, 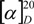 = *–*59.9 (c, 0.5, EtOH), *de* = 96%.; (–)-**1**.HCl salt mp 192–194 °C, 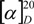 = *–*14.8 (c, 0.6, EtOH), *ee* = 96%. The ^1^H- and ^13^C-NMR spectroscopic and elemental analysis data on the enantiomeric derivative were in accordance with those for racemic (±)-**1**·HCl [26].

*Ethyl (1R*,*2S*,*3R*,*4S)-3-aminobicyclo[2.2.2]oct-5-ene-2-carboxylate* (+)-**1**. (+)-**1**·(+)DBTA salt, mp 189–190 °C, 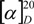 = +63.5 (c, 0.5, EtOH), *de* = 98%.; (+)-**1**.HCl salt, mp 192–194 °C, 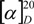 = +15.3 (c, 0.6, EtOH), *ee* = 98%. The ^1^H- and ^13^C-NMR spectroscopic and elemental analysis data on the enantiomeric derivative were in accordance with those for racemic (±)-**1**·HCl [26].

### 3.4. Isomerization of Amino Esters (–)-**1** and (+)-**1**

Freshly prepared NaOEt (280 mg, 4 mmol) was added to a solution of the free base of (–)-**1** or (+)-1 (394 mg, 2 mmol) in dry EtOH (12 mL), and the mixture was heated for 2 h at 70 °C. It was then concentrated under reduced pressure, taken up in CH_2_Cl_2_ and washed with H_2_O (2 × 20 mL). The combined organic phase was dried (Na_2_SO_4_), the solvent was evaporated off, the residue was dissolved in EtOH containing 22% HCl (2 mL), and the solution was stirred for 10 min at room temperature. After removal of the solvent, amino ester hydrochloride **2** was obtained, which was recrystallized from EtOH/Et_2_O.

*Ethyl (1S*,*2S*,*3S*,*4R)-3-aminobicyclo[2.2.2]oct-5-ene-2-carboxylate hydrochloride* (+)-**2**. White crystals (256 mg, 55%), mp 220 °C (with sublimation), 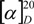 = +47.9 (c, 0.5, EtOH), *ee* = 98%. ^1^H-NMR: δ = 0.98–1.09 (m, 1H, H-8), 1.18–1.26 (t, *J* = 7.1 Hz, 3H, CH_3_), 1.18–1.38 (m, 3H, H-7, H-7, H-8,), 2.282.32 (m, 1H, H-2), 2.82–2.91 (m, 2H, H-1, H-4), 3.64 (t, *J* = 3.2 Hz, 1H, H-3), 4.8–4.23 (m, 2H, OCH_2_), 6.18 (t, *J* = 7.1Hz, 1H, H-5) 6.53 (t, *J* = 7.5 Hz 1H, H-6) 7.95 (bs, 3H, NH_3_^+^) ppm. ^13^C-NMR: δ = 14.9, 19.9, 23.0, 33.3, 33.4, 49.3, 51.7, 61.6, 130.9, 136.6, 172.4 ppm. Anal. Calcd for C_11_H_18_ClNO_2_ (231,74): C, 57.02; H, 7.83; Cl 15,30; N, 6,04. Found: C, 56.81; H, 8.04; N, 6.05.

*Ethyl (1R*,*2R*,*3R*,*4S)-3-aminobicyclo[2.2.2]oct-5-ene-2-carboxylate hydrochloride* (–)-**2**. White crystals (263 mg, 56%), mp 178–180 °C, 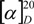 = *–*43 (c, 0.4, EtOH), *ee* = 98%. The ^1^H- and ^13^C-NMR spectroscopic data on the enantiomeric derivative were consistent with the data on the antipode (+)-**2**. Anal. Calcd for C_11_H_18_ClNO_2_ (231.74): C, 57.02; H, 7.83; Cl 15.30; N, 6.04. Found: C, 56.94; H, 8.11; N, 6.12.

### 3.5. General Procedure for Hydrogenation of Amino Esters (–)-1 (+)-1, (–)-2 and (+)-2 over Pd/C Catalyst in a Fixed-Bed Reactor

Free amino ester (–)-**1**, (+)-**1**, (–)-**2** or (+)-**2** (98 mg, 5 mmol) was dissolved in dry EtOH (100 mL) to form a 0.05 M solution. This solution was hydrogenated in the ThalesNano H-cube™ system, using a 10% Pd/C catalyst at a flow rate of 1 mL min^−1^ at 50 °C and a H_2_ pressure of 50 bar. The EtOH was removed under vacuum. The residue was dissolved in EtOH containing 22% HCl (2 mL) and the solution was stirred for 10 min at room temperature. After removal of the solvent, the amino ester hydrochloride was obtained.

*Ethyl (2S*,*3S)-3-aminobicyclo[2.2.2]octane-2-carboxylate Hydrochloride* (–)-**3**. An oil (90 mg, 91%), 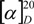 = *–*17.5 (c, 1.0, MeOH), *ee* = 99%. ^1^H-NMR: δ = 1.21 (t, *J* = 7.2, 3H, CH_3_) 1.25–1.86 (m, 9H, H-5, H-5, H-6, H-6, H-7, H-7, H-8, H-8, H-2), 1.90–1.95 (m, 1H, H-4), 2.54–2.58 (m, 1H, H-4), 3.54–3,64 (m, 1H, H-3), 4.06–4.21 (m, 2H, OCH_2_), 8.16 (bs, 3H, NH_3_^+^) ppm. ^13^C-NMR: δ = 14.7, 18.6, 21.0, 24.5, 25.3, 27.8, 28.8, 47.9, 50.5, 61.58, 166.4 ppm. Anal. Calcd for C_11_H_20_ClNO_2_ (233.74): C, 56.52; H, 8.62; Cl 15.17; N, 5.99. Found: C, 56.21; H, 8.39; N, 5.93.

*Ethyl (2R*,*3R)-3-aminobicyclo[2.2.2]octane-2-carboxylate Hydrochloride* (+)-**3**. An oil (90 mg, 91%), 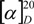 = +15,4 (c, 0.9, MeOH), *ee* = 99%. The ^1^H- and ^13^C-NMR spectroscopic data on the enantiomeric derivative were consistent with the data on the antipode (–)-**3**. Anal. Calcd for C_11_H_20_ClNO_2_ (233.74): C, 56.52; H, 8.62; Cl 15.17; N, 5.99. Found: C, 56.29; H, 8.88; N, 6.14.

*Ethyl (2R*,*3S)-3-aminobicyclo[2.2.2]octane-2-carboxylate Hydrochloride* (–)-**4**. White crystals (86 mg, 86%,), mp 195-196 °C, 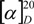 = *–*31.5 (c, 0.6, EtOH), *ee* = 99%. ^1^H-NMR: δ = 1.22 (t, *J* = 7.1, 3H, CH_3_) 1.29–1.98 (m, 10H, H-5, H-5, H-6, H-6, H-7, H-7, H-8, H-8, H-1, H-4), 3.00 (dd, *J* = 5.1, *J* = 1.1 H-2), 3.47 (d, *J* = 4.6 1H, H-3), 4.03–4.23 (m, 2H, OCH_2_), 8.00 (bs, 3H, NH_3_^+^) ppm. ^13^C-NMR: δ = 14.9, 18.7, 21.2, 24.5, 25.2, 28.0, 28.7, 43.4, 48.8, 61.4, 172.8 Anal. Calcd for C_11_H_20_ClNO_2_ (233.74): C, 56.52; H, 8.62; Cl 15.17; N, 5.99. Found: C, 56.76; H, 8.49; N, 6.17. The *ee* values for esters (–)-**4** and (+)-**4** were determined after derivatization with Ac_2_O in the presence of DMAP and pyridine (1:9) by using Perkin-Elmer GC instrumentation equipped with CP-Chirasil L-Val columns (30 m) with the temperature held at 140 °C for 5 min and then raised to 160 °C at 10 °C min^−1^, flow rate: 1 mL min^−1^, retention times: (–)-**4**: 9.62 min; (+)-**4**: 9.97 min.

*Ethyl (2S*,*3R)-3-aminobicyclo[2.2.2]octane-2-carboxylate Hydrochloride* (+)-**4**. White crystals (81 mg, 81%,), mp 193–194°C, 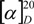 = +30.1 (c, 0.4, EtOH) *ee* = 99%. The ^1^H- and ^13^C-NMR spectroscopic data on the enantiomeric derivative were consistent with the data on the antipode (–)-**4**. Anal. Calcd for C_11_H_20_ClNO_2_ (233.74): C, 56.52; H, 8.62; Cl 15.17; N, 5.99. Found: C, 56.81; H, 8.75; N, 6.21.

### 3.6. General Procedure for Hydrolyses of Amino Esters **1**–**4**

The individual amino ester enantiomers **1**–**4** (198 mg, 1 mmol) were dissolved in water (4 mL) in a 10 mL pressurized reaction vial, and the solution was stirred at 100 °C for 60 min at max. 150 W microwave irradiation. The solvent was evaporated off, and the crude amino acid was recrystallized from H_2_O/acetone to afford a white crystalline product.

*(1S*,*2S*,*3S*,*4R)-3-Aminobicyclo[2.2.2]oct-5-ene-2-carboxylic Acid* (+)-**5**. White crystals (80 mg, 46%), mp 245–260 °C (dec.), 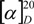 = +51 (c, 0.3, H_2_O). ^1^H-NMR: δ = 0.95–1.55 (m, 4H, H-7, H-7, H-8, H-8), 2.13–2.21 (m, 1H, H-2), 2.75–2.85 (m, 1H, H-1) 2.85–2.95 (m, 1H, H-4), 3.63 (t, *J* = 3.0 Hz, 1H, H-3), 3.76 (bs, 3H, NH_3_^+^) 6.17 (t, *J* = 7.1 Hz, 1H, H-5) 6.52 (t, *J* = 7.4 Hz 1H, H-6) ppm. ^13^C-NMR: δ = 20.0, 23.2, 33.3, 33,6, 49.9, 52.0, 130.9, 136.8, 173.9 ppm. Anal. Calcd for C_9_H_13_NO_2_ (167.09): C, 64.65; H, 7.84; N, 8.38. Found: C, 64.71; H, 7.98; N, 8.45.

*(1R*,*2R*,*3R*,*4S)-3-Aminobicyclo[2.2.2]oct-5-ene-2-carboxylic Acid* (–)-**5**. White crystals (96 mg, 56%), mp 270–277 °C (dec.), 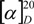 = *–*64 (c, 0.5, H_2_O). The ^1^H- and ^13^C-NMR spectroscopic data on the enantiomeric derivative are consistent with the data of the antipode (+)-**5**. Anal. Calcd for C_9_H_13_NO_2_ (167.09): C, 64.65; H, 7.84; N, 8.38. Found: C, 64.83; H, 7.99; N, 8.61.

*(2S*,*3S)-3-Aminobicyclo[2.2.2]octane-2-carboxylic Acid* (–)-**6**. White crystals (116 mg, 67%), mp 255–260 °C (dec.), 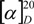 = *–*35.4 (c, 0.5, H_2_O). ^1^H-NMR: δ = 1.39–1.75 (m, 8H, H-5, H-5, H-6, H-6, H-7, H-7, H-8, H-8), 1.87–1.91 (m, 1H, H-4), 2.07–2.11 (m, 1H, H-1) 2.34 (d, *J* = 6.1, 1H, H-2), 3.90 (d, *J* = 6.1 Hz, 1H, H-3). ^13^C-NMR: δ = 18.4, 20.9, 24.2, 25.5, 28.1, 29.2, 51.0, 52.9, 180.9 ppm. Anal. Calcd for C_9_H_15_NO_2_ (169.11): C, 63.88; H, 8.93; N, 8.28. Found: C, 64.07; H, 9.11; N, 8.08.

*(2R*,*3R)-3-Aminobicyclo[2.2.2]octane-2-carboxylic Acid* (+)-**6**. White crystals (100 mg, 58%), mp 255–260 °C (dec.), 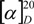 = +35.1 (c, 0.4, H_2_O). The ^1^H- and ^13^C-NMR spectroscopic data on the enantiomeric derivative were consistent with the data on the antipode (–)-**6**. Anal. Calcd for C_9_H_15_NO_2_ (169.11): C, 63.88; H, 8.93; N, 8.28. Found: C, 64.12; H, 9.05; N, 8.41.

*(1S*,*2R*,*3S*,*4R)-3-Aminobicyclo[2.2.2]oct-5-ene-2-carboxylic Acid* (–)-**7**. White crystals (96 mg, 56%), mp 215–219 °C (dec.), 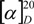 = *–*3 (c, 0.4, H_2_O). The ^1^H- and ^13^C-NMR spectroscopic and elemental analysis data on the enantiomeric derivative were in accordance with those for racemic (±)-**7** [29].

*(1R*,*2S*,*3R*,*4S)-3-Aminobicyclo[2.2.2]oct-5-ene-2-carboxylic Acid* (+)-**7**. White crystals (88 mg, 51%), mp 220–221 °C (dec.), 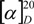 = +3.2 (c, 0.4, H_2_O) The ^1^H- and ^13^C-NMR spectroscopic and elemental analysis data on the enantiomeric derivative were in accordance with those for racemic (±)-**7** [29].

*(2R*,*3S)-3-Aminobicyclo[2.2.2]octane-2-carboxylic Acid* (–)-**8**. White crystals (92 mg, 54%), mp 223–230 °C (dec.), 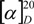 = *–*49 (c, 0.3, H_2_O). The ^1^H- and ^13^C-NMR spectroscopic and elemental analysis data on the enantiomeric derivative were in accordance with those for racemic (±)-**8** [29].

*(2S*,*3R)-3-Aminobicyclo[2.2.2]octane-2-carboxylic Acid* (+)-**8**. White crystals (83 mg, 48%), mp 236–240 °C (dec.), 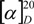 = +46.5 (c, 0.3, H_2_O). The ^1^H- and ^13^C-NMR spectroscopic and elemental analysis data on the enantiomeric derivative were in accordance with those for racemic (±)-**8** [29].

### 3.7. Syntheses of Ureas (+)-**9** and (–)-**10**

Amino ester base (–)-**1** or (–)-**2** (100 mg, 0.33 mmol) was dissolved in dry Et_2_O (20 mL) and a 10% excess of (*S*)-(–)-1-phenylethyl isocyanate (50 mg, 0.34 mmol) was added. The mixture was allowed to stand for 24 h at room temperature. After evaporation, the resulting crystalline urea adducts were recrystallized from iPr_2_O.

*Ethyl (1S*,*2R*,*3S*,*4R)-3-[3-(1-(S)-phenylethyl)ureido]bicyclo[2.2.2]oct-5-ene-2-carboxylate* (+)-**9**. White crystals (92 mg, 82%), mp 195–200 °C, 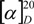 = +8 (c, 0.3, EtOH). ^1^H-NMR: δ = 0.99–1.07 (m 1H, H-7) 1.11–1.20 (m, 1H, H-7) 1.16 (t, *J* = 7.2, 3H, CH_3_) 1.23 (d, *J* = 7.1 (3H, CH_3_) 1.43–1.53 (m, 2H, H-8), 2.44 (m, 1H, H-1) 2.66 (m, 1H, H-4) 2.96 (d, *J* = 9.8, 1H, H-2) 3.90–4.06 (m, 2H, OCH_2_) 4.3 (td, *J* = 10.1, *J* = 1.5, 1H, H-3) 4.68 (m, 1H, CH) 5.34 (d, *J* = 10.4 H, NH) 6.12 (t, *J* = 7.2, 1H, H-5, 6.47 (t, *J* = 7.4, 1H, H-6) 6.54 (d, *J* = 8.1 1H, NH) 7.16–7.32 (m, 5H, Ar) ^13^C-NMR: δ = 14.8, 22.7, 24.4, 25.3, 32.6, 36.9, 49.3, 51.3, 51.4, 60.4, 126.5, 127.2, 129.0, 130.5, 137.1, 146.6, 157.0, 173.0. Anal. Calcd for C_20_H_26_N_2_O_3_ (342.43): C 70.15, H 7.65; N 8.18. Found C 70.22, H 7.92, N 8.12.

*Ethyl (1R*,*2R*,*3R*,*4S)-3-[3-(1-(S)-phenylethyl)ureido]bicyclo[2.2.2]oct-5-ene-2-carboxylate* (–)-**10**. White crystals (108 mg, 96%), a white solid, mp. 130–135 °C, 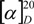 = *–*101 (c, 0.2, EtOH) ^1^H-NMR: δ = 0.92–1.06 (m 1H, H-7) 1.19–1.23 (m, 1H, H-7) 1.16 (t, *J* = 7.1, 3H, CH_3_) 1.28 (d, *J* = 6.9 (3H, CH_3_) 1.36–1.52 (m, 2H, H-8), 1.95 (m, 1H, H-2) 2.58 (m, 1H, H-1) 2.70 (m, 1H, H-4) 4,08–4.23 (m, 3H, OCH_2_, H-3) 4.64 (m, 1H, CH) 5.54 (d, *J* = 7.9 H, NH) 6.12–6,21 (m, 2H, H-5, NH), 6.44 (t, *J* = 7.3, 1H, H-6) 7.14–7.35 (m, 5H, Ar) ^13^C-NMR: δ = 14.9, 20.3, 23.3, 24.3, 33.6, 36.0, 49.3, 51.5, 53.1, 60.8, 126.6, 127.3, 129.1, 132.8, 135.7, 146.5, 157.3, 173.8, Anal. Calcd for C_20_H_26_N_2_O_3_ (342.43): C 70.15, H 7.65, N 8.18. Found C 70.31, H 7.79, N 8.27.

### 3.8. Racemic Compounds

All the reactions were first optimized for the racemic compounds. The ^1^H- and ^13^C-NMR spectroscopic and elemental analysis data on the racemic derivatives were in accordance with those for enantiomers.

*Data on racemates* (±)–**2**-(±)-**8**

*Ethyl 3-endo-aminobicyclo[2.2.2]oct-5-ene-2-exo-carboxylate hydrochloride* (±)-**2**: White crystals, mp 227–229 °C.

*Ethyl trans-3-aminobicyclo[2.2.2]octane-2-carboxylate hydrochloride* (±)-**3**: White crystals, mp 130–133 °C.

*Ethyl cis-3-aminobicyclo[2.2.2]octane-2-carboxylate hydrochloride* (±)-**4**: White crystals, mp 189–191 °C.

*3-endo-Aminobicyclo[2.2.2]oct-5-ene-2-exo-carboxylic acid* (±)-**5**: White crystals, mp 250–255 °C.

*trans-3-Aminobicyclo[2.2.2]octane-2-carboxylic acid* (±)-**6**: White crystals, mp 255–260 °C.

*2,3-Diendo-3-aminobicyclo[2.2.2]oct-5-ene-2-carboxylic acid* (±)-**7**: White crystals, mp 228–231 °C, Lit. mp 204–208 °C [29].

*cis-3-Aminobicyclo[2.2.2]octane-2-carboxylic acid* (±)-**8**: White crystals, mp 208–210, Lit. mp 232–235 °C [32].

### 3.9. X-Ray Crystallographic Studies

The crystallographic data on compounds (+)-9 and (–)-10 were collected at 123 K with an Agilent SuperNova dual wavelength diffractometer equipped with an Atlas CCD area detector with the use of Cu-Kα radiation and the CrysAlisPro program package [33]. The empirical absorption correction was performed with the SCALE3 ABSPACK scaling algorithm as implemented in the CrysAlisPro program [33]. The crystal data along with selected refinement details for compounds (+)-**9** and (–)-**10** are presented in Table 2.

The structures were solved by direct methods with the SHELXS-97 [34] program or the SIR-97 [35] program, and the full-matrix least squares refinements on F^2^ were performed with the SHELXL-9728 program. Figures were drawn with Diamond 3 [36]. For all compounds, the heavy atoms were refined anisotropically. The CH hydrogen atoms were included at the calculated distances with fixed displacement parameters from their host atoms (1.2 or 1.5 times the host atom). The NH hydrogens were located from the electron density map and refined isotropically. The absolute configurations of the compounds were determined from the Flack parameters.

Detailed crystallographic data for compound (+)-**9** (CCDC 918507) and (–)-**10** (CCDC 918508) is deposited at the Cambridge Crystallographic Data Centre. These data can be obtained free of charge via http://www.ccdc.cam.ac.uk/conts/retrieving.html (or from the CCDC, 12 Union Road, Cambridge CB2 1EZ, UK; Fax: +44 1223 336033; E-mail: deposit@ccdc.cam.ac.uk).

**Table 2:** Crystallographic data for compounds (+)-**9** and (–)-**10**.

Compound	(+)-9	( *–*)-10
Formula	C_20_H_26_N_2_O_3_	C_20_H_26_N_2_O_3_
Mr	342.43	342.43
Crystal system	orthorhombic	orthorhombic
Space group (no.)	P2_1_2_1_2_1_(19)	P2_1_2_1_2_1_ (19)
a (Å)	12.0973(2)	8.9562(3)
b (Å)	17.4189(2)	18.4189(6)
c (Å)	17.5471(2)	22.9915(3)
α (°)	90	90
β (°)	90	90
γ (°)	90	90
V (Å)	3697.55(9)	3792.75(18)
Z	8	8
Dc (g cm^−3^)	1.230	1.199
T (K)	123	123
μ(CuK_α_)	0.665	0.648
Observed reflections	6269	6726
R_int_	0.0279	0.0213
Parameters	467	477
Flack’s parameter	0.05(17)	0.0(2)
R_1_^a^	0.0406 (0.0386) ^b^	0.0521 (0.0480)^b^
wR_2_^c^	0.0991 (0.0969)	0.1266 (0.1230)

^a^ R_1_ = ∑||Fo| − |Fc||/∑|Fo|; ^b^ Values in parentheses for reflections with I > 2σ(I); ^c^ wR_2_ = {∑[w(F2o − F2c)2]/∑[w(F2o)2]}½ and w = 1/[σ2(F2o) + (aP)2 + (bP)], where P = (2F2c + F2o)/3.

## 4. Conclusions

In conclusion, we have successfully synthetized all four enantiomers of 2,3-*diendo*-3-aminobicyclo[2.2.2]oct-5-ene-2-carboxylic acid and 3-*endo*-aminobicyclo[2.2.2]oct-5-ene-2-*exo*-carboxylic acid and their saturated analogues in a simple scalable protocol. The prepared compounds are highly likely to be useful and well applicable in synthetic chemistry and drug research, with the creation of diverse libraries of conformationally constrained compounds.

## References

[1] Konishi M., Nishio M., Saitoh K., Miyaki T., Oki T., Kawaguchi H. (1989). Cispentacin: A new antifungal antibiotic I. Production, isolation, physico-chemical properties and structure. J. Antibiot..

[2] Oki T., Hirano M., Tomatsu K., Numata K., Kamei H. (1989). Cispentacin, a new antifungal antibiotic II. *In vitro* and *in vivo* antifungal activities. J. Antibiot..

[3] Kuhl A., Hahn M.G., Dumić M., Mittendorf J. (2005). Alicyclic β-amino acids in medicinal chemistry. Amino Acids.

[4] Pou A., Moyano A. (2013). Stereoselective organocatalytic approach to α,β-disubstituted β-amino acids: A sort enantioselective synthesis of Cispentacin. Eur. J. Org. Chem..

[5] Martinek T.A., Fülöp F. (2012). Peptidic foldamers: Ramping up diversity. Chem. Soc. Rev..

[6] Horne W.S. (2011). Peptide and peptoid foldamers in medicinal chemistry. Expert Opin. Drug Disc..

[7] Sathe M., Thavaselvam D., Srivastava A.K., Kaushik M.P. (2008). Synthesis and antimalarial evaluation of cyclic β-amino acid-containing dipeptides. Molecules.

[8] Keresztes A., Szücs M., Borics A., Kövér K.E., Forró E., Fülöp F., Tömböly C., Péter A., Páhi A., Fábián G. (2008). New endomorphin analogues containing alicyclic β-amino acids: Influence on bioactive conformation and pharmacological profile. J. Med. Chem..

[9] Horne W.S., Gellman S.H. (2008). Foldamers with heterogeneous backbones. Acc. Chem. Res..

[10] Seebach D., Gardiner J. (2008). β-Peptidic peptidomimetics. Acc. Chem. Res..

[11] Martín-Vilà M., Muray E., Aguado G.P., Alvarez-Larena A., Branchadell V., Minguillón C., Giralt E., Ortuňo R.M. (2000). Enantioselective synthetic approaches to cyclopropane and cyclobutane β-amino acids: Synthesis and structural study of a conformationally constrained β-dipeptide. Tetrahedron-Asymmetr..

[12] Gauzy C., Pereira E., Faure S., Aitken D.J. (2004). Synthesis of (+)-(1*S*,2*R*) and (–)-(1*R*,2*S*)-2-aminocyclobutane-1-carboxylic acids. Tetrahedron Lett..

[13] Fernandez C., Pereira E., Faure S., Aitken D.J. (2009). Expedient preparation of all isomers of 2-aminocyclobutanecarboxylic acid in enantiomerically pure form. J. Org. Chem..

[14] Forró E., Fülöp F. (2007). The first direct enzymatic hydrolysis of alicyclic β-amino esters: A route to enantiopure *cis* and *trans* β-amino acids. Chem. Eur. J..

[15] Priego J., Flores P., Ortiz-Nava C., Escalante J. (2004). Synthesis of enantiopure *cis*- and *trans*-2-aminocyclohexane-1-carboxylic acids from octahydroquinazolin-4-ones. Tetrahedron- Asymmetr..

[16] Forró E., Fülöp F. (2008). Vapour-assisted enzymatic hydrolysis of β-lactams in a solvent-free system. Tetrahedron-Asymmetr..

[17] Palkó M., Benedek G., Forró E., Wéber E., Hänninen M., Sillanpää R. (2010). Synthesis of mono- and dihydroxy-substituted 2-aminocyclooctanecarboxylic acid enantiomers. Tetrahedron-Asymmetr..

[18] Bolm C., Schiffers I., Atodiresei I., Hackenberger P.R. (2003). An alkaloid-mediated desymmetrization of *meso*-anhydrides via a nucleophilic ring opening with benzyl alcohol and its application in the synthesis of highly enantiomerically enriched β-amino acids. Tetrahedron-Asymmetr..

[19] Forró E., Fülöp F. (2004). Synthesis of enantiopure 1,4-ethyl- and 1,4-ethylene-bridged cispentacin by lipase-catalyzed enantioselective ring opening of β-lactams. Tetrahedron-Asymmetr..

[20] Todorov P.T., Wesselinova D.W., Pavlov N.D., Martinez J., Calmes M., Naydenova E.D. (2012). Cytotoxic activity of new racemic and optically active *N*-phosphonoalkyl bicyclic β-amino acids against human malignant cell lines. Amino Acids.

[21] André C., Calmes M., Escale F., Amblard M., Martinez J., Songis O. (2012). New 1,3-amino alcohols derived from enantiopure bridgehead β-aminobicyclo[2.2.2]oct-5-ene-2-carboxylic acids. Amino Acids.

[22] Calmes M., Escale F., Didierjean C., Martinez J. (2011). Synthesis of enantiopure trans-N-Boc-3-aminobicyclo[2.2.2]octane-2-carboxylic acids and their bicyclic 1,3-amino alcohol derivatives via the [4+2] cycloaddition of 1,3-cyclohexadiene to a chiral β-nitroacrylate. Chirality.

[23] Ruebsam F., Murphy D.E., Tran C.V., Li L.S., Zhao J., Dragovich P.S., McGuire H.M., Xiang A.X., Sun Z., Ayida B.K. (2009). Discovery of tricyclic 5,6-dihydro-1*H*-pyridin-2-ones as novel, potent, and orally bioavailable inhibitors of HCV NS5B polymerase. Bioorg. Med. Chem. Lett..

[24] Bunue E., Cativiela C., Diaz-de-Villages M.D., Galvez J.A. (1996). A new conformationally restricted aspartic acid analogue with a bicyclo[2.2.2]octanes skeleton. Acta Cryst. C.

[25] Pope W.J., Peachey S.J. (1899). The application of powerful optically active acids to the resolution of externally compensated basic substances. Resolution of tetrahydroquinaldine. J. Chem. Soc. Trans..

[26] Sakurai R., Yuzawa A., Sakai K. (2008). Practical resolution of 3-aminopyrrolidine via diastereomeric salt formation with (*S*)-2-methoxy-2-phenylacetic acid. Tetrahedron-Asymmetr..

[27] Faigl F., Fogassy E., Nógrádi M., Pálovics E., Schindler J. (2008). Strategies in optical resolution: A practical guide. Tetrahedron-Asymmetr..

[28] Leeman M., Kaptein B., Kellogg R.M. (2009). Nucleation inhibition in attrition-enhanced Pope-Peachey type of diastereomeric resolutions. Tetrahedron-Asymmetr..

[29] Palkó M., Sohár P., Fülöp F. (2011). Synthesis and transformations of di-*endo*-3-aminobicyclo-[2.2.2]oct-5-ene-2-carboxylic acid derivatives. Molecules.

[30] Fogassy E., Lopata A., Faigl F., Darvas F., Ács M., Tőke L. (1980). A quantitative approach to optical resolution. Tetrahedron Lett..

[31] Forró E. (2009). New gas chromatographic method for the enantioseparation of β-amino acids by a rapid double derivatization technique. J. Chromatogr. A.

[32] Moriconi E.I., Crawford W.C. (1968). Reaction of chlorosulfonyl isocyanate with bridge bi- and tricyclic olefins. J. Org. Chem..

[33] Agilent (2011). CrysAlis PRO.

[34] Sheldrick G.M. (2008). A short history of SHELX. Acta Crystallogr. A.

[35] Altomare A., Burla M.C., Camalli M., Cascarano G.L., Giacovazzo C., Guagliardi A., Moliterni A.G.G., Polidori G., Spagna R. (1999). SIR97: A new tool for crystal structure determination and refinement. J. Appl. Cryst..

[36] Pennington W.T. (1999). DIAMOND-visual crystal structure information system. J. Appl. Cryst..

